# Rotation Covariant Image Processing for Biomedical Applications

**DOI:** 10.1155/2013/931507

**Published:** 2013-04-18

**Authors:** Henrik Skibbe, Marco Reisert

**Affiliations:** ^1^Graduate School of Informatics, Kyoto University, Gokasho, 611-0011 Uji, Kyoto, Japan; ^2^Department of Diagnostic Radiology, Medical Physics, University Medical Center, Breisacher Street 60a, 79106 Freiburg, Germany

## Abstract

With the advent of novel biomedical 3D image acquisition techniques, the efficient and reliable analysis of volumetric images has become more and more important. The amount of data is enormous and demands an automated processing. The applications are manifold, ranging from image enhancement, image reconstruction, and image description to object/feature detection and high-level contextual feature extraction. In most scenarios, it is expected that geometric transformations alter the output in a mathematically well-defined manner. In this paper we emphasis on 3D translations and rotations. Many algorithms rely on intensity or low-order tensorial-like descriptions to fulfill this demand. This paper proposes a general mathematical framework based on mathematical concepts and theories transferred from mathematical physics and harmonic analysis into the domain of image analysis and pattern recognition. Based on two basic operations, spherical tensor differentiation and spherical tensor multiplication, we show how to design a variety of 3D image processing methods in an efficient way. The framework has already been applied to several biomedical applications ranging from feature and object detection tasks to image enhancement and image restoration techniques. In this paper, the proposed methods are applied on a variety of different 3D data modalities stemming from medical and biological sciences.

## 1. Introduction

 The analysis of three-dimensional images has gained more and more importance in recent years. Particular in the medical and biological sciences, new acquisition techniques lead to an enormous amount of 3D data calling for automated analysis. In this paper, we show how the harmonic analysis of the 3D rotation group offers a convenient and computationally efficient framework for rotation covariant image processing and analysis. Most of the state-of-the-art techniques rely on “low”-order features such as intensities, gradients of intensities, or second order tensors like the Hessian matrix or the structure tensor [1]. For example, consider a lesion detection/segmentation problem in a *T*_1_-weighted magnetic resonance image. A typical procedure for solving such a task would rely on a local image feature extraction step such as the computation of a Laplacian- or a Gaussian-pyramid. Once the feature images are computed, a healthy group of volunteers is used to determine the distribution of such features for subjects in a healthy condition.

From such a distribution, we can estimate the probabilities for the absence or presence of lesions in a voxel-by-voxel manner. Instead of solely using 0-order features, such as the Laplacian-pyramid, higher order tensor fields can be used to derive further scalar valued quantities. Such features can be the smoothed intensity gradient magnitudes (1-order features), or the eigenstructures of a Hessian matrix field or a structure tensor field (2-order features). However, due to their mathematical and computational complexity, features of order three or even higher order are rarely used. This paper proposes a unified framework that can cope with high-order features in a systematic way. The proposed framework is based on the harmonic, irreducible representations of the 3D rotation group. This guarantees the most sparse tensor representation. Consequently, in comparison with ordinary Cartesian tensor analysis, the algorithms and the handling are operationally clearer and more efficient.

 Given a Cartesian tensor *t*_*i*_1_,…,*i*_*n*__ of rank *n*, such a tensor can be the result from a simple projection onto moment functions or from a differentiation process (for instance, a gradient is a tensor of order 1, and a Hessian matrix is a tensor of oder 2). A tensor can be considered as a feature describing an object in a rotation covariant way; that is, if the original object is rotated by a rotation matrix **R**, the tensor rotates in the following manner:

(1) $$\begin{array}{c} {\left(gt\right)}_{i_{1},\ldots ,i_{n}}=\sum_{j_{1},\ldots ,j_{n}}R_{i_{1},j_{1}}\cdots R_{i_{n},j_{n}}t_{j_{1},\ldots ,j_{n}}, \end{array}$$

where *g* denotes an element of the 3D rotation group. A tensor rotation is a common operation in many applications, for instance, steering a local image descriptor (a tensor) with respect to some data dependent reference frame. From a computationally point of view a Cartesian tensor rotation is quite inconvenient. Typically, there are symmetries with respect to index permutations (for instance, the Hessian matrix is a symmetric tensor). These symmetries have to be taken into account to provide an efficient computation. Another problem is that the tensor rotation matrix *R*_*i*_1_,*j*_1__ ⋯ *R*_*i*_*n*_,*j*_*n*__ is “full”; that is, all elements *t*_*j*_1_,…,*j*_*n*__ mix under rotations. Spherical tensor analysis, where tensors appear in their irreducible representations, solves these problems, and, even more, it offers further advantages regarding tensor operations. Suppose that we aim at extracting rotation invariant features. Given a Cartesian tensor *t*, for Cartesian tensors, the basic operation is *tensor contraction*. The tensor is contracted down to a rank 0 tensor by repeatedly combining two indexes (with the Kronecker delta *δ*_*ij*_), or three indexes (with the *ϵ*-tensor *ϵ*_*ijk*_). This can be done in several ways, for example, linearly ∑_*ii*_ *t*_·,*i*,·,*i*,·_, quadratically ∑_*ij*_ *t*_·,*i*,·_ *t*_·,*i*,·_, or even cubicly ∑_*ijk*_ *ϵ*_*ijk*_ *t*_·,*i*,·_ *t*_·,*j*,·_ *t*_·,*k*,·_. It is possible to combine different tensors as well. A problem that occurs is ambiguities; in the presence of symmetries, some ways might end up in the same result, some may not. In contrast, the spherical tensor analysis offers a systematic way of performing such operations. The Kronecker and *ϵ* products are replaced by one single *spherical product* which allows for multiplying spherical tensors of arbitrary rank. The even parity products are related to the Kronecker product, the odd parity products to *ϵ* products. 

In this paper, we want to review the basics of spherical tensor analysis and how it can be applied to image processing problems. In Section 2, we introduce the basic concepts such as the notion of a spherical tensors. We define the spherical product and introduce its properties. We also show how spherical tensors are related to ordinary Cartesian tensors. In Section 3, the so-called spherical tensor derivative operators (shortly spherical derivatives) are introduced. The spherical derivative operators are able to connect spherical tensor fields of different rank. We discuss several properties and derive their representation in polar coordinates. We focus on two types of basis systems evolving from the spherical differentiation process: the Gauss-Laguerre functions and the spherical Gabor-functions. Both are known to be very important in pattern analysis. The differential relationships of these functions offer an efficient way to compute projections onto these type of functions. In Section 4, expansions in terms of tensorial harmonics are discussed, which are just the straight-forward generalization of ordinary scalar-valued spherical harmonic expansions. Finally, in Section 5, several biomedical applications are reviewed and discussed.

## 2. Spherical Tensor Analysis

 Let **D**_*g*_^*j*^ be the unitary irreducible representation of a *g* ∈ *SO*(3) of order *j* with *j* ∈ *ℕ*. They are also known as the *Wigner D-matrices* (e.g., see [3]). The representation **D**_*g*_^*j*^ acts on a vector space *V*_*j*_ which is represented by *ℂ* ^2*j*+1^. We write the elements of *V*_*j*_ in bold face, for example, **u** ∈ *V*_*j*_, and write the 2*j* + 1 components in unbolt face *u*_*m*_ ∈ *ℂ* where *m* = −*j*,…, *j*. For the transposition of a vector/matrix, we write **u** ^*T*^; the joint complex conjugation and transposition is denoted by $u^{⊤}={\bar{u}}^{T}$. In this terms, the unitarity of **D**_*g*_^*j*^ is expressed by the formula (**D**_*g*_^*j*^)^*⊤*^ **D**_*g*_^*j*^ = **I**.

Note that we treat the space *V*_*j*_ as a real vector space of dimensions 2*j* + 1, although the components of **u** may be complex. This means that the space *V*_*j*_ is only closed under weighted superpositions with real numbers. As a consequence of this, we always have that the components are interrelated by $\bar{u_{m}}={\left(-1\right)}^{m}u_{-m}$. From a computational point of view, this is an important issue. Although the vectors are elements of *ℂ* ^2*j*+1^, we just have to store just 2*j* + 1 real numbers.

We denote the standard basis of *ℂ* ^2*j*+1^ by **e**_*m*_^*j*^, where the *n*th component of **e**_*m*_^*j*^ is *δ*_*mn*_. In contrast, the standard basis of *V*_*j*_ is written as **c**_*m*_^*j*^ = ((1 + **i**)/2)**e**_*m*_^*j*^ + (−1)^*m*^((1 − **i**)/2)**e**_−*m*_^*j*^. We denote the corresponding “imaginary” space by **i** *V*_*j*_; that is, elements of **i** *V*_*j*_ can be written as **i** **v** where **v** ∈ *V*_*j*_. So, elements **w** ∈ **i** *V*_*j*_ fulfill $\bar{w_{m}}={\left(-1\right)}^{m+1}w_{-m}$. Hence, we can write the space *ℂ* ^2*j*+1^ as the direct sum of the two spaces *ℂ* ^2*j*+1^ = *V*_*j*_ ⊕ **i** *V*_*j*_. The standard coordinate vector **r** = (*x*, *y*, *z*)^*T*^ ∈ ℝ^3^ has a natural relation to elements **u** ∈ *V*_1_ by

(2) u=x−y2c11+zc01−x+y2c−11=(12(x−iy)z−12(x+iy))=Sr∈V1.

Note that **S** is an unitary coordinate transformation. The representation **D**_*g*_^1^ is directly related to the real-valued rotation matrix **U**_*g*_ ∈ *SO*(3) ⊂ ℝ^3×3^ by **D**_*g*_^1^ = **S** **U**_*g*_ **S** ^*⊤*^.

**Definition 1**

A function **f** : ℝ^3^ ↦ *V*_*j*_ is called a spherical tensor field of rank *j* if it transforms with respect to rotations as

(3) $$\begin{array}{c} \left(gf\right)\left(r\right)∶=D_{g}^{j}f\left(U_{g}^{⊤}r\right), \end{array}$$

for all *g* ∈ *SO*(3). The space of all spherical tensor fields of rank *j* is denoted by *𝒯*_*j*_.

### 2.1. Spherical Tensor Coupling

 Now, we define a family of bilinear forms that connect tensors of different ranks.

**Definition 2**

For every *j* ≥ 0, we define a family of bilinear forms of type

(4) $$\begin{array}{c} \circ_{j}:V_{j_{1}}\times V_{j_{2}}\mapsto {\mathbb{C}}^{2j+1}, \end{array}$$

where *j*_1_, *j*_2_ ∈ *ℕ* has to be chosen according to the triangle inequality |*j*_1_ − *j*_2_ | ≤*j* ≤ *j*_1_ + *j*_2_. It is defined by

(5) $$\begin{array}{c} {\left(e_{m}^{j}\right)}^{⊤}\left(v\;\circ_{j}\;w\right)∶=\sum_{m=m_{1}+m_{2}}\langle jm\;|\;j_{1}m_{1},j_{2}m_{2}\rangle v_{m_{1}}w_{m_{2}}, \end{array}$$

where 〈*jm* | *j*_1_ *m*_1_, *j*_2_ *m*_2_〉 are the Clebsch-Gordan coefficients.

The characterizing property of these products is that they respect the rotations of the arguments.

**Proposition 3**

Let **v** ∈ *V*_*j*_1__ and **w** ∈ *V*_*j*_2__; then, for any *g* ∈ *SO*(3),

(6) $$\begin{array}{c} \left(D_{g}^{j_{1}}v\right)\circ_{j}\left(D_{g}^{j_{2}}w\right)=D_{g}^{j}\left(v\;\circ_{j}\;w\right) \end{array}$$

holds.

**Proof**

The components of the left-hand side look as

(7) (emj)⊤((Dgj1v)∘j(Dgj2w)) =∑m=m1+m2m1′m2′〈jm ∣ j1m1,j2m2〉Dm1m1′j1Dm2m2′j2vm1′wm2′.

First one has to insert the identity by using orthogonality relation ((B.1)) with respect to *m*_1_′ and *m*_2_′. Then, we can use relation ((C.2)) and the definition of ∘_*j*_ to prove the assertion.

**Proposition 4**

If *j*_1_ + *j*_2_ + *j* is even, then ∘ is symmetric, otherwise antisymmetric. The spaces *V*_*j*_ are closed for the symmetric product, and for the antisymmetric product this is not the case. Consider

(8) $$\begin{array}{c} j+j_{1}+j_{2}\;is\;\;even\Rightarrow v\;\circ_{j}\;w\in V_{j},\\ j+j_{1}+j_{2}\;is\;\;odd\Rightarrow v\;\circ_{j}\;w\in iV_{j}, \end{array}$$

where **v** ∈ *V*_*j*_1__ and **w** ∈ *V*_*j*_2__.

**Proof**

The proposition is proved by the symmetry properties of the Clebsch-Gordan coefficients ((B.6)). To show the closure property, consider

(9) (emj)⊤v ∘j w¯  =∑m=m1+m2〈jm ∣ j1m1,j2m2〉vm1¯wm2¯  =∑m=m1+m2(−1)m〈jm ∣ j1m1,j2m2〉v−m1w−m2  =∑m=m1+m2(−1)m+j+j1+j2      ×〈j(−m) ∣ j1m1,j2m2〉vm1wm2  =(−1)m+j+j1+j2(e−mj)⊤v ∘j w¯.

Hence, we have for even *j* + *j*_1_ + *j*_2_ the “realness” condition complying to *V*_*j*_ and for odd *j* + *j*_1_ + *j*_2_ the “imaginariness” condition for **i** *V*_*j*_, which prove the statements.

We will later see that the symmetric product plays an important role, in particular, because we can normalize it in an special way such that it shows a more gentle behavior with respect to the spherical harmonics.

**Definition 5**

For every *j* ≥ 0 with |*j*_1_ − *j*_2_ | ≤*j* ≤ *j*_1_ + *j*_2_ and even *j* + *j*_1_ + *j*_2_, we define a family of symmetric bilinear forms by

(10) $$\begin{array}{c} v\;{•}_{j}\;w∶=\frac{1}{\langle j0\;|\;j_{1}0,j_{2}0\rangle }v\;\circ_{j}\;w. \end{array}$$

For the special case *j* = 0, the arguments have to be of the same rank due to the triangle inequality. Actually in this case, the symmetric product coincides with the standard inner product

(11) $$\begin{array}{c} v\;{•}_{0}\;w=\sum_{m=-j}^{m=j}{\left(-1\right)}^{m}v_{m}w_{-m}=w^{⊤}v, \end{array}$$

where *j* is the rank of **v** and **w**.

The introduced product can also be used to combine tensor fields of different rank by point-wise multiplication.

**Proposition 6**

Let **v** ∈ *𝒯*_*j*_1__ and **w** ∈ *𝒯*_*j*_2__ and *j* chosen such that |*j*_1_ − *j*_2_ | ≤*j* ≤ *j*_1_ + *j*_2_; then,

(12) $$\begin{array}{c} f\left(r\right)=v\left(r\right)\circ_{j}\;w\left(r\right) \end{array}$$

is in *𝒯*_*j*_, that is, a tensor field of rank *j*.

In fact, there is another way to combine two tensor fields: by convolution. The advantage of the convolution is that the evolving product also is covariant with respect to translation; that is, the product is covariant to 3D Euclidean motion.

**Proposition 7**

Let **v** ∈ *𝒯*_*j*_1__ and **w** ∈ *𝒯*_*j*_2__ and *j* chosen such that |*j*_1_ − *j*_2_ | ≤*j* ≤ *j*_1_ + *j*_2_; then,

(13) $$\begin{array}{c} \left(v\;{\tilde{\circ }}_{j}\;w\right)\left(r\right)∶=\int_{{\mathbb{R}}^{3}}v\left(r^{\prime }-r\right)\;\circ_{j}\;w\left(r^{\prime }\right)dr^{\prime } \end{array}$$

is in *𝒯*_*j*_, that is, a tensor field of rank *j*.

Given a translation *τ*, the following two relations hold:

(14) $$\begin{array}{c} \left(\tau v\right)\circ_{j}\left(\tau w\right)=\tau \left(v\;\circ_{j}\;w\right),\\ v\;{\tilde{\circ }}_{j}\left(\tau w\right)=\left(\tau v\right){\tilde{\circ }}_{j}\;w=\tau \left(v\;{\tilde{\circ }}_{j}\;w\right). \end{array}$$

Further important properties of the products are their associativity rules.

**Proposition 8**

The product ∘ is associative as

(15) $$\begin{array}{c} v^{j_{1}}\circ_{\ell }\left(w^{j_{2}}\circ_{j_{2}+j_{3}}y^{j_{3}}\right)=\left(v^{j_{1}}\circ_{j_{1}+j_{2}}w^{j_{2}}\right)\circ_{\ell }y^{j_{3}} \end{array}$$

holds if *j*_1_ + *j*_2_ + *j*_3_ = *ℓ*. And

(16) $$\begin{array}{c} v^{j_{1}}\circ_{\ell }\left(w^{j_{2}}\circ_{j_{2}-j_{3}}y^{j_{3}}\right)=\left(v^{j_{1}}\circ_{j_{2}-j_{1}}w^{j_{2}}\right)\circ_{\ell }y^{j_{3}} \end{array}$$

holds if *ℓ* = *j*_2_ − (*j*_1_ + *j*_3_) ≥ 0. And

(17) $$\begin{array}{c} v^{j_{2}}\circ_{\ell }\left(w^{j_{1}}\circ_{j_{1}+j_{3}}y^{j_{3}}\right)=\left(v^{j_{1}}\circ_{j_{2}-j_{1}}w^{j_{2}}\right)\circ_{\ell }y^{j_{3}} \end{array}$$

with *ℓ* = *j*_2_ − (*j*_1_ + *j*_3_) ≥ 0.

### 2.2. Spherical and Solid Harmonics

 Due to their special properties, the spherical harmonics (see, Appendix A for definition) play the central role in spherical tensor analysis. One of the most important ones is that each **Y** ^*j*^, interpreted as a tensor field of rank *j*, is a fix-point with respect to rotations; that is,

(18) $$\begin{array}{c} \left(gY^{j}\right)\left(r\right)=D_{g}^{j}Y^{j}\left(U_{g}^{⊤}r\right)=Y^{j}\left(r\right). \end{array}$$

Consequently,

(19) $$\begin{array}{c} Y^{j}\left(U_{g}r\right)=D_{g}^{j}Y^{j}\left(r\right). \end{array}$$

The **Y** ^*j*^ form an orthogonal and complete basis of the functions defined on the 2-sphere. Hence, any real square-integrable scalar field *f* ∈ *𝒯*_0_ can be written as

(20) $$\begin{array}{c} f\left(r\right)=\sum_{j=0}^{\infty }a^{j}{\left(r\right)}^{⊤}Y^{j}\left(r\right)=\sum_{j=0}^{\infty }\;\sum_{m=-j}^{m=j}\bar{a_{m}^{j}}\left(r\right)Y_{m}^{j}\left(r\right). \end{array}$$

**Figure 1:**
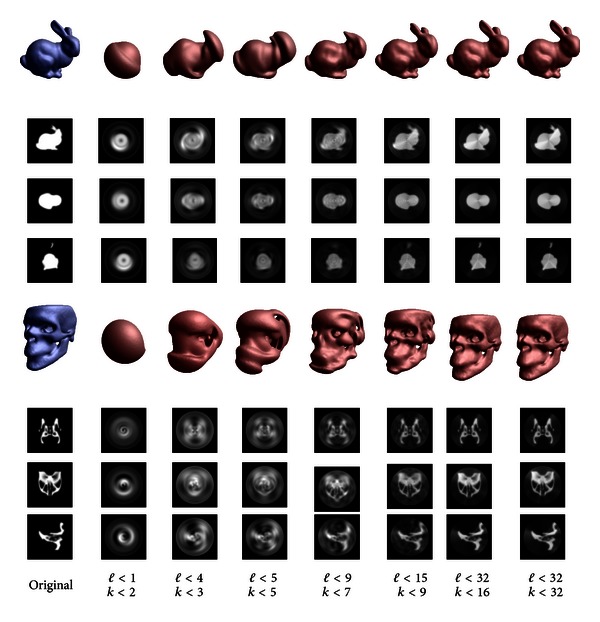
A spherical harmonic decomposition of images can be seen as some kind of frequency decomposition. A band limited expansion of a volumetric images is illustrated. We see that lower frequency components (right-hand side) are roughly representing the important characteristics of the objects. However, higher frequency components are necessary to represent the details. For the expansion here, we use a Fourier-like basis for representing the images in radial direction. Here, *ℓ* represents the order of the spherical harmonics and *k* the number of radial frequency components taken into account. The image shows an isosurface rendering together with the centered *X*,  *Y*, and *Z*-slice. The interested reader is referred to [2].

A band-limited spherical harmonic representation of two images is illustrated in Figure 1.

The expansion coefficients of the rotated function (*gf*)(**r**) = *f*(**U**_*g*_^*⊤*^ **r**) are simply **D**_*g*_^*j*^ **a** ^*j*^(*r*), which can be concluded from the fix-point property. In the following, we always use Racah's normalization (also known as semi-Schmidt normalization); that is,

(21) 〈Ymj,Ym′j′〉=∫S2Ymj(s)  Ym′j′¯(s)ds=4π2j+1δjj′δmm′,

where the integral ranges over a sphere using the standard measure. With this, the coupling of two spherical harmonics gives, again, a spherical harmonic

(22) $$\begin{array}{c} Y^{j_{1}}\left(r\right){•}_{j}Y^{j_{2}}\left(r\right)=Y^{j}\left(r\right). \end{array}$$

From a computational perspective, this property can be used to efficiently compute higher order harmonics for lower ones.

Besides the spherical harmonics, the so-called solid harmonics, often appear in the context of harmonic analysis of the 3D rotation group. They are the homogeneous solutions of the Laplace-equation and are just related by **R** ^*j*^ : = *r* ^*j*^ **Y** ^*j*^, and they are homogeneous polynomials of degree *j*; that is, **R** ^*j*^(*λ* **r**) = *λ* ^*j*^ **R** ^*j*^(**r**).

### 2.3. Relation to Cartesian Tensors

 The correspondence of spherical and Cartesian tensors of rank 0 is trivial. For rank 1, it is just the matrix **S** that connects the real-valued vector **r** ∈ ℝ^3^ with the spherical coordinate vector **u** = **S** **r** ∈ *V*_1_. For rank 2, the consideration gets more intricate. Consider a real-valued Cartesian rank-2 tensor **T** ∈ ℝ^3×3^ and the following unique decomposition:

(23) T=(t00t01t02t10t11t12t20t21t22)=αI+Tanti+Tsym,

where *α* ∈ ℝ, **T**_anti_ is an antisymmetric matrix, and **T**_sym_ a traceless symmetric matrix. In fact, this decomposition follows the same manner as the spherical tensor decomposition. A rank 0 spherical tensor corresponds to the identity matrix in Cartesian notation, while the rank 1 spherical tensor to a antisymmetric 3 × 3 matrix or, equivalently, to a vector. And finally, the rank 2 spherical tensor corresponds to a traceless, symmetric matrix. So, let us consider the spherical decomposition. For convenience, let **T** ^*s*^ = **S** **T** **S** ^*⊤*^; then, the components of the corresponding spherical tensors **b** ^*j*^ with *j* = 0,1, 2 are

(24) $$\begin{array}{c} b_{m}^{j}=\sum_{m_{1}+m_{2}=m}\langle 1m_{1},1m_{2}\;|\;jm\rangle {\left(-1\right)}^{m_{2}}T_{m_{1}\left(-m_{2}\right)}^{s}, \end{array}$$

where **b** ^0^ corresponds to *α*, **b** ^1^ to **T**_anti_ and **b** ^2^ to **T**_sym_. Explicitly, the relation to **T** is

(25) $$\begin{array}{c} b^{0}=\frac{-\left(t_{00}+t_{11}+t_{22}\right)}{\sqrt{3}},\\ b^{1}=\left(\begin{array}{c} \frac{1}{2}\left(t_{20}-t_{02}+i\left(t_{21}-t_{12}\right)\right)\\ \frac{i}{\sqrt{2}}\left(t_{10}-t_{01}\right)\\ \frac{1}{2}\left(t_{20}-t_{02}-i\left(t_{21}-t_{12}\right)\right) \end{array}\right),\\ b^{2}=\left(\begin{array}{c} \frac{1}{2}\left(t_{00}-t_{11}+i\left(t_{01}+t_{10}\right)\right)\\ \frac{1}{2}\left(\left(t_{02}+t_{20}\right)+i\left(t_{12}+t_{21}\right)\right)\\ \frac{-1}{\sqrt{6}}\left(t_{00}+t_{11}-2t_{22}\right)\\ \frac{1}{2}\left(-\left(t_{02}+t_{20}\right)+i\left(t_{12}+t_{21}\right)\right)\\ \frac{1}{2}\left(t_{00}-t_{11}-i\left(t_{01}+t_{10}\right)\right) \end{array}\right). \end{array}$$

The inverse of this “Cartesian to spherical”-transformation is

(26) $$\begin{array}{c} T_{m_{1}m_{2}}^{s}=\sum_{j=0,2}\;\sum_{m=-j}^{m=j}\langle 1m_{1},1\left(-m_{2}\right)\;|\;jm\rangle {\left(-1\right)}^{m_{2}}b_{m}^{j}. \end{array}$$

Note that for arbitrary ranked Cartesian tensor, the relations are not that trivial.

## 3. Spherical Derivatives

This section proposes the concepts of differentiation in the context of spherical tensor analysis. First, we will introduce the spherical derivative operator which connects spherical tensor fields of different ranks by differentiation. The basic idea is simple; formally replace the coordinates **r** = (*x*, *y*, *z*) appearing within the solid harmonics **R** ^*j*^ by the gradient operator (∂_*x*_, ∂_*y*_, ∂_*z*_).

**Proposition 9 (spherical derivatives)**

Let **f** ∈ *𝒯*_*ℓ*_ be a tensor field. The spherical up-derivative ∇^1^ : *𝒯*_*ℓ*_ → *𝒯*_*ℓ*+1_ and the down-derivative ∇_1_ : *𝒯*_*ℓ*_ → *𝒯*_*ℓ*−1_ are defined as

(27) $$\begin{array}{c} \nabla^{1}f∶=R^{1}\left(\nabla \right){•}_{\ell +1}f,\\ \nabla_{1}f∶=R^{1}\left(\nabla \right){•}_{\ell -1}f, \end{array}$$

where ∇ is the gradient operator (∂_*x*_, ∂_*y*_, ∂_*z*_).

In fact there are much more rotation covariant differential operators than the two defined previously. Given a tensor field **f**, any field of the form **g** = **R** ^*j*^(∇)•_*ℓ*_ **f**, which we obtain via differentiation, is a spherical tensor field, too. But the up- and down-derivatives are from a computational point very attractive, because, as shown earlier, they allow an iterative computation of higher order differentials, which is computationally much more efficient than the direct way. For further discussion on the spherical tensor derivative operator, consider the spherical derivatives in the Fourier domain, where they act by point-wise •-multiplications with a solid harmonic **i** *k* **Y** ^1^(**k**) = **i** **R** ^1^(**k**) = **i** **S** **k** where *k* = |**k**| is the frequency magnitude.

**Proposition 10 (Fourier representation)**

Let $\tilde{f}(k)$ be the Fourier transformation of some **f** ∈ *𝒯*_*ℓ*_ and $\tilde{\nabla }\;$representations of the spherical derivative in the Fourier domain that are implicitly defined by $\tilde{\left(\nabla f\right)}=\tilde{\nabla }\;\tilde{f}$; then,

(28) $$\begin{array}{c} {\tilde{\nabla }}^{1}\tilde{f}\left(k\right)=iR^{1}\left(k\right){•}_{\ell +1}\tilde{f}\left(k\right), \end{array}$$

(29) $$\begin{array}{c} {\tilde{\nabla }}_{1}\tilde{f}\left(k\right)=iR^{1}\left(k\right){•}_{\ell -1}\tilde{f}\left(k\right). \end{array}$$

**Proof**

See [4].

Both statements are direct consequences of the Fourier correspondences for the ordinary partial derivatives. For scalar fields, we can generalize this statement also for higher orders.

**Proposition 11 (multiple spherical derivatives)**

For *n* ≥ *i*, he defines ∇_*i*_^*n*^ : *𝒯*_0_ → *𝒯*_*n*−*i*_ by

(30) $$\begin{array}{c} \nabla_{i}^{n}∶=\;\nabla_{i}\nabla^{n}∶=\underset{i\text{-times}}{\underset{︸}{\;\nabla_{1}\cdots \;\nabla_{1}}}\underset{n\text{-times}}{\underset{︸}{\;\nabla^{1}\cdots \nabla^{1}}}. \end{array}$$

In the Fourier domain, these multiple derivatives act by

(31) $$\begin{array}{c} \left({\tilde{\nabla }}_{i}^{n}\tilde{f}\right)\left(k\right)={\left(i\right)}^{n+i}\;R_{i}^{n}\left(k\right)\tilde{f}\left(k\right). \end{array}$$

Using this one can show that ∇_*i*_^*n*^ = ∇^*n*−*i*^Δ^*i*^, where Δ is the Laplace operator.

**Proof**

See [5].

We want to emphasize that both statements only hold for scalar-valued fields, and generalizations to tensor-valued do not hold in general due to the nontrivial associativity rules.

**Proposition 12 (product rule)**

Let **f** ∈ *𝒯*_*ℓ*_ and *h* ∈ *𝒯*_0_; then, one has the product rules

(32) $$\begin{array}{c} \nabla^{1}\left(hf\right)=\;\nabla^{1}h{•}_{\ell +1}f+h\nabla^{1}f,\\ \nabla_{1}\left(hf\right)=\;\nabla^{1}h{•}_{\ell -1}f+h\nabla_{1}f. \end{array}$$

It is well known that convolutions commute with differentiation, and actually there are generalized commutation rules for spherical tensor fields.

**Proposition 13 (commuting property for convolutions)**

Let **f** ∈ *𝒯*_*k*_ and **g** ∈ *𝒯*_*j*_ be arbitrary spherical tensor fields; then,

(33) $$\begin{array}{c} \left(\nabla^{\ell }f\right){\tilde{•}}_{J}\;g=f\;{\tilde{•}}_{J}\left(\nabla_{\ell }\;g\right), \end{array}$$

(34) $$\begin{array}{c} \left(\nabla^{\ell }f\right){\tilde{•}}_{L}\;\;g=f\;{\tilde{•}}_{L}\left(\nabla^{\ell }g\right), \end{array}$$

where *J* = *j* − (*ℓ* + *k*) and *L* = *j* + *ℓ* + *k*.

**Proof**

Both assertions are founded by the associativity of the spherical product. Consider the first statement in the Fourier domain by using (28) and then apply the associativity given in (17) as follows:

(35) (∇ℓ~f~)•Jg~=(R1•k+ℓ(∇ℓ−1~f~))•Jg~  =(∇ℓ−1~f~)•J(R1•j−1g~)=(∇ℓ−1~f~)•j(∇~1g~),

where we abbreviated **R** ^1^ = **R** ^1^(**i** **k**). A repeated application of this proves the first assertion. For the second statement, it is similar but using the associativity as given in (15).

This proposition shows again the importance of the up- and down-derivatives. For general derivative operators **R** ^*j*^(∇)•_*ℓ*_ **f**, the previous commutations rules do not hold. The previous convolution property is of particular importance for the efficient covariant processing of 3D images. The major motivation is to compute convolutions with the spherical harmonic basis in an efficient way. Suppose that the goal is to compute

(36) $$\begin{array}{c} f=\left(R^{j}e^{-r^{2}/2}\right)*g, \end{array}$$

where *g* is some arbitrary scalar image. In fact, as we will show in the next section, one can show that ∇^*j*^ *e* ^−*r*^2^/2^ = (−1)^*j*^ **R** ^*j*^ *e* ^−*r*^2^/2^. Together with the convolution theorem, we get

(37) f=(Rje−r2/2)∗g=(−1)j∇j(e−r2/2∗g)

which enables us to compute the convolution by an repeated application of the spherical derivatives, which is computationally much cheaper than a direct convolution (even by the use of the Fast Fourier Transform).

### 3.1. Spherical Derivatives in Polar Representation

 To get a better understanding of what happens during the differentiation via spherical derivatives, we consider their properties in polar representations.

**Lemma 14**

Given a spherical tensor field **f** ^*j*^ ∈ *𝒯*_*j*_ whose angular and radial component are separable such that **f** ^*j*^(**r**) = **Y** ^*j*^(**r**)*f* ^*j*^(*r*), where *f* ^*j*^ : ℝ → *ℂ* denotes the function representing the radial component of **f** ^*j*^, then the spherical up- and down-derivatives of **f** ^*j*^ can be computed by

(38) $$\begin{array}{c} \left(\nabla^{1}f^{j}\right)\left(r\right)=Y^{j+1}\left(r\right)r^{j}\frac{\partial }{\partial r}\frac{1}{r^{j}}f^{j}\left(r\right), \end{array}$$

(39) $$\begin{array}{c} \left(\nabla_{1}f^{j}\right)\left(r\right)=Y^{j-1}\left(r\right)\frac{1}{r^{j+1}}\frac{\partial }{\partial r}r^{j+1}f^{j}\left(r\right), \end{array}$$

respectively.

**Proof**

See [7].

### 3.2. Gauss-Laguerre Functions

Previously, we already stated that ∇^*j*^ *e* ^−*r*^2^/2^ = (−1)^*j*^ **R** ^*j*^ *e* ^−*r*^2^/2^ holds; in fact, there is a more general statement involving the so-called Laguerre polynomials. This offers the possibility to compute convolutions with the evolving functions in an iterative and efficient way. We denote by *L*_*n*_^*α*^ the *α* associated Laguerre polynomial of order *n* ((F.1)). We further denote by

(40) $$\begin{array}{c} L_{n}^{j}\left(r\right)∶=R^{j-n}\left(r\right)L_{n}^{\left(j-n)+(1/2\right)}\left(\frac{r^{2}}{2}\right) \end{array}$$

the spherical tensor valued polynomials *L*_*n*_^*j*^ ∈ *𝒯*_*j*−*n*_. These polynomials are widely known as Laguerre Gaussian-type functions in the field of theoretical chemistry (e.g., see [8] or [9]). In the image processing community, these functions are known as generic neighborhood operators [10] and are used, for example, for key-point detection [11].

**Theorem 15**

The Gaussian windowed polynomials *L*_*n*_^*j*^(**r**)*e* ^−*r*^2^/2^ can be computed iteratively in terms of ∇_*n*_^*j*^ starting with an isotropic Gaussian; namely,

(41) $$\begin{array}{c} L_{n}^{j}\left(r\right)e^{-r^{2}/2}=\frac{{\left(-1\right)}^{j}}{n!2^{n}}\nabla_{n}^{j}e^{-r^{2}/2}\;. \end{array}$$

**Proof**

See [7].

### 3.3. Gabor Functions

 Gabor functions, that is, Gaussian-windowed plane waves, play an important role in image processing due to the fact that the different frequency components of signals can be studied locally. This information is, for example, used for tracking [12] or feature extraction [6]. Thus, it is of particular interest to provide efficient methods to apply Gabor filters. One way is to explicitly represent a finite number of Gabor kernels, each representing a certain orientation of the plane-wave [13]. The problem is that the orientation space must be discretized. However, representing Gabor functions in terms of spherical derivatives offers a way to compute Gabor filter responses for the whole range of possible orientations. First, note that applying spherical derivatives on a plane wave gives a quite neat result as

(42) $$\begin{array}{c} \nabla^{j}e^{ik^{⊤}r}={\left(i\right)}^{j}R^{j}\left(k\right)e^{ik^{⊤}r}. \end{array}$$

Following the proof from Section 3.1, a similar result holds for the spherical Bessel function, which constitutes the radial part in the harmonic expansion of the plane wave as

(43) ∇jj0(kr)=(k)jYj(r)jj(kr)=(k)jBj(r,k).

In the following, we show that there exists a very similar way to represent the Gaussian windowed wave in terms of the derivatives of the Gaussian windowed Bessel functions. Let

(44) $$\begin{array}{c} B_{s}^{0}\left(r,k\right)∶=j_{0}\left(kr\right)e^{-r^{2}/\left(2s\right)} \end{array}$$

be the Gaussian windowed 0-order Bessel functions. The parameter *s* ∈ ℝ_*s*>0_ represents the size of the Gaussian window with respect to the wave. With (38) and ((D.3)), we can derive the higher order Gaussian windowed Bessel functions *B*_*s*_^*j*^ : = (−1)^*j*^∇^*j*^ *B*_*s*_^0^.

**Theorem 16**

The spherical derivatives *B*_*s*_^*j*^ of the Gaussian windowed 0-ordered Bessel functions *B*_*s*_^0^ are given by

(45) $$\begin{array}{c} B_{s}^{j}\left(r,k\right)=Y^{j}\left(r\right)\left[\sum_{i=0}^{j}\left(\begin{array}{c} j\\ i \end{array}\right){\left(\frac{r}{s}\right)}^{j-i}{\left(k\right)}^{i}j_{i}\left(kr\right)\right]e^{-r^{2}/2s}\;. \end{array}$$

Consider that *B*_*s*→*∞*_^*j*^ = *B* ^*j*^. The Gabor wave can now be represented by a superposition of Bessel functions *B*_*s*_^*j*^, each representing a certain angular frequency; namely,

(46) eikTre−r2/2s≈∑j(i)jαj(k)Bsj(r,k)•0Yj(k)=∑j(−i)jαj(k)∇jBs0(r,k)•0Yj(k),

where *α*_*j*_(*k*) ∈ ℝ are real-valued weighting factors.

**Proof**

See [7].

## 4.  Tensorial Harmonic Expansions

 In most image processing applications, the data to be processed is of scalar nature; that is, for each voxel, we observe one single intensity value. But there are actually acquisition techniques, where the measurement itself is already a tensorial quantity. For example, in diffusion weighted magnet resonance imaging (DW-MRI), rank 2 tensors are common. Or, in phase contrast MRI velocity, vectors are measured. Thus, there is a great interest to represent these measurement in an appropriate way. In [14], we proposed to expand a spherical tensor field **f** ∈ *𝒯*_*ℓ*_ of rank *ℓ* as follows:

(47) $$\begin{array}{c} f\left(r\right)=\sum_{j=0}^{\infty }\;\sum_{k=-\ell }^{k=\ell }a_{k}^{j}\left(r\right)\circ_{\ell }Y^{j}\left(r\right), \end{array}$$

where **a**_*k*_^*j*^(*r*) ∈ *𝒯*_*j*+*k*_ are expansion coefficients. For *ℓ* = 0, the expansion coincides with the ordinary scalar spherical harmonic expansion. We can observe properties very similar to the ordinary SH expansion; that is,

(48) (gf)(r)=Dgℓf(Ug⊤r)=∑j=0∞ ∑k=−ℓk=ℓDgj+kakj(r)∘ℓYj(r).  

A rotation of the tensor field affects the expansion coefficients **a**_*k*_^*j*^ to be multiplied from the left with **D**_*g*_^*j*+*k*^. So, the previous expansion shows the same, very convenient, rotation behavior like an SH expansion, which can be used, for example, to extract invariant local descriptors in a simple way. And in fact, the previous representation is orthogonal and complete. By setting **a**_*k*_^*j*^(*r*) = ∑_*m*=−(*j*+*k*)_^*m*=*j*+*k*^ *a*_*km*_^*j*^(*r*)**e**_*m*_^*j*+*k*^, we can identify the functional basis **Z**_*km*_^*j*^ as

(49) $$\begin{array}{c} f\left(r\right)=\sum_{j=0}^{\infty }\;\sum_{k=-\ell }^{k=\ell }\;‍\sum_{m=-\left(j+k\right)}^{m=j+k}a_{km}^{j}\left(r\right)\underset{Z_{km}^{j}}{\underset{︸}{e_{m}^{j+k}\circ_{\ell }Y^{j}\left(r\right)}}. \end{array}$$

**Proposition 17 (tensorial harmonics)**

The functions **Z**_*km*_^*j*^ : *S* ^2^ ↦ *V*_*ℓ*_ provide a complete and orthogonal basis of the angular part of *𝒯*_*ℓ*_, that is;

(50) $$\begin{array}{c} \int_{S^{2}}{\left(Z_{km}^{j}\left(s\right)\right)}^{⊤}Z_{k^{\prime }m^{\prime }}^{j^{\prime }}\left(s\right)ds=\frac{4\pi }{N_{j,k}}\delta_{j,j^{\prime }}\delta_{k,k^{\prime }}\delta_{m,m^{\prime }}, \end{array}$$

where

(51) $$\begin{array}{c} N_{j,k}=\frac{1}{2\ell +1}\left(2j+1\right)\left(2\left(j+k\right)+1\right). \end{array}$$

The functions **Z**_*km*_^*j*^ are called the tensorial harmonics.

### 4.1. Symmetric Tensor Fields

 In this section, we discuss the properties of expansion coefficients of specific tensor fields, expanded in terms of tensorial harmonics. We show that symmetries in a tensor field are simplifying the tensorial harmonic expansion coefficients. This is similar to the ordinary spherical harmonic expansion. For example, the point symmetry *f*(**r**) = *f*(−**r**) of a scalar fields leads to vanishing spherical harmonic coefficients for odd *j*. In the following, we consider similar symmetries for tensorial harmonics.

The rotation symmetry of a spherical tensor field **f** ∈ *𝒯*_*ℓ*_ around the *z*-axis is expressed algebraically by the fact that *g*_*ϕ*_ **f** = **f** for all rotation *g*_*ϕ*_ around the *z*-axis. Such fields can easily be obtained by averaging a general tensor field **f** over all these rotations as

(52) $$\begin{array}{c} f_{s}=\frac{1}{2\pi }\int_{0}^{2\pi }g_{\phi }f\;d\phi . \end{array}$$

It is well known that the representation **D**_*g*_*ϕ*__^*j*^ of such a rotation is diagonal; namely, *D*_*g*_*ϕ*_,*mm*′_^*j*^ = *δ*_*mm*′_ *e* ^**i***mϕ*^. Hence, the expansion coefficients *a*_*km*_^*j*^ of **f**_*s*_ vanish for all *m* ≠ 0. Thus, we can write any rotation symmetric tensor field as

(53) $$\begin{array}{c} f_{s}\left(r\right)=\sum_{j=0}^{\infty }\;\sum_{k=-\ell }^{k=\ell }a_{k}^{j}\left(r\right)\;e_{0}^{j+k}\circ_{\ell }Y^{j}\left(r\right). \end{array}$$

We call such a rotation symmetric field torsion-free if *g*_*yz*_ **f**_*s*_ = **f**_*s*_, where *g*_*yz*_ ∈ *O*(3) is a reflection with respect to the *yz*-plane (or *xz*-plane). The action of such a reflection on spherical tensors is given by *D*_*g*_*yz*_,*mm*′_^*j*^ = (−1)^*m*^ *δ*_*m*(−*m*′)_. Similar to the rotational symmetry, we can obtain such fields by averaging over the symmetry operation as

(54) $$\begin{array}{c} f_{\text{stf}}=\frac{1}{2}\left(f_{s}+g_{yz}f_{s}\right). \end{array}$$

Note that the mirroring operation for a spherical harmonic is just a complex conjugation; that is, $Y^{j}(U_{g_{yz}}^{T}r)=\bar{Y^{j}}(r)$. The consequence for (53) is that all terms where the *k* + *ℓ* are odd vanish. The reason for that is mainly Proposition 4 because with its help we can show that

(55) Dgyzℓ(e0j+k∘ℓYj(UgyzTr))=(−1)(k+ℓ)(e0j+k∘ℓYj(r))

holds.

Finally, consider the reflection symmetry with respect to the *xy*-plane. This symmetry is particularly important for fields of even rank. The symmetry is algebraically expressed by *g*_*xy*_ **f**_*s*_ = **f**_*s*_ where *g*_*xy*_ ∈ *O*(3) is a reflection with respect to the *xy*-plane, whose action on spherical tensors is given by *D*_*g*_*yz*_,*mm*′_^*j*^ = (−1)^*j*^ *δ*_*mm*′_. Averaging over this symmetry operation has the consequence that expansion terms with odd *j* are vanishing. For odd rank tensor fields, the reflection symmetry is not imperative. But there is typically an antisymmetry of the form *g*_*xy*_ **f**_*s*_ = −**f**_*s*_. This antisymmetry lets the expansion terms vanish with even index *j*.

## 5. Applications

 In the context of rotation covariant image processing, the applications of the proposed framework are manifold. The mathematical representation might appear unfamiliar, but the provided tools can be used quite easily. Basically, there are two types of operations: differentiation by spherical tensor derivatives and multiplication by spherical tensor products. The spherical derivatives can be used in two ways. On the one hand, the up-derivatives can be used to “create” new tensor fields out of existing fields by incorporating neighborhood relations. This can be regarded as a simple and efficient way to compute local meaningful image descriptors in a covariant way. On the other hand, the down-derivatives can be used to gather information from a local point neighborhood and form a lower ranked tensor field via superposition. Due to the tensorial nature, the information is able to interfere in a destructive or constructive way. The spherical products are the basic nonlinear ingredient in the framework. They can be used to combine tensor fields in a nonlinear, covariant manner.

Several principles in the image processing and pattern recognition [15–17] literature are based on the following principle: compute, in a first step, local descriptors at several image locations, make some inference based on this knowledge, and cast this information back by combining evidence from several locations. In fact, our framework is ideally suited to adopt this principle. First, local descriptors are densely computed by differentiation for *all* image locations. Then, the information is combined by using spherical products in a nonlinear and nontrivial way. Finally, we use again the spherical derivative to form neighborhood descriptors. The descriptors are then used for object or feature detection.

In the following, we give examples of the proposed framework in several application domains.

### 5.1. Implementation

**Figure 2:**
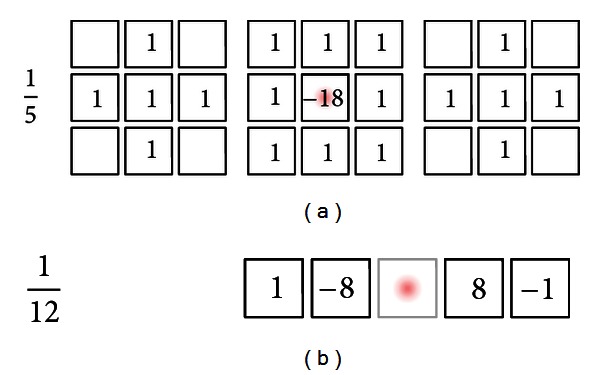
The discrete differential operators we use for realizing the discrete spherical derivative operators. On the left-hand side, the corresponding global weights are depicted. The red dot denotes the current image position.

**Figure 3:**
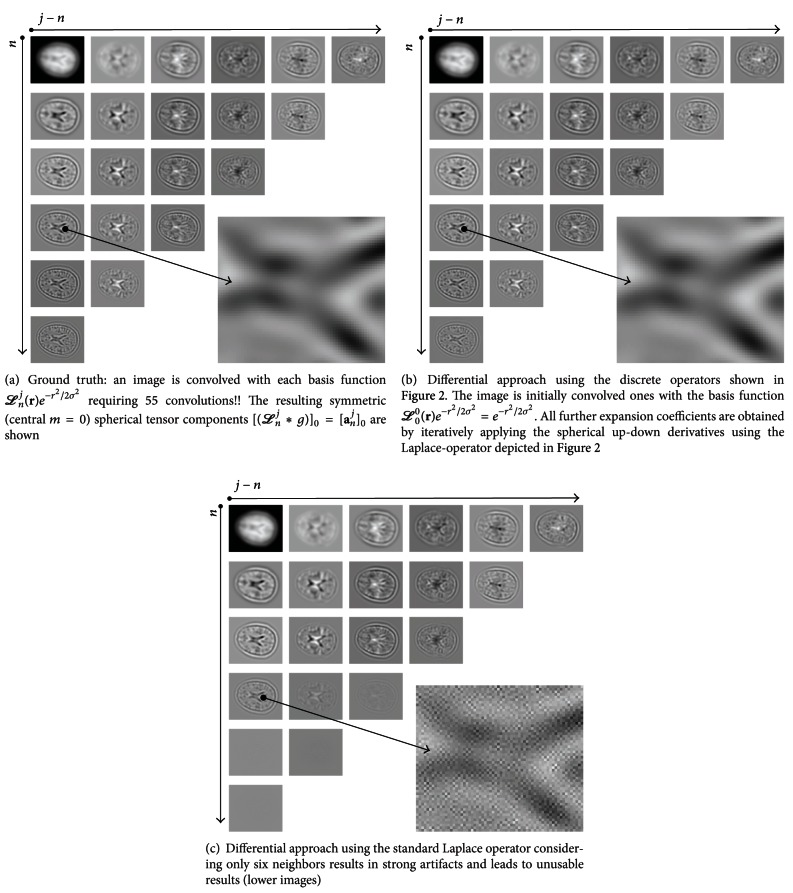
The theory in practice: Laguerre expansion of a volumetric image with *j* + *n* ≤ 5 and a Gaussian width of *σ* = 6. For the experiments we use an image (size 144 × 224 × 256) showing the *T*_1_-weighted MRT image of a human skull. In (a) we depict the center slice of the 3D volume showing the real-valued parts (*m* = 0) of the expansion coefficients computed explicitly by convolution of the image with the kernel functions (${\left[a_{n}^{j}\right]}_{m}(x)=(g*{\left[\bar{L_{n}^{j}}\right]}_{m}e^{-r^{2}/2})(x)$). (b) Shows the same expansion coefficients obtained when using the proposed differential approach, with ${\left[a_{n}^{j}\right]}_{m}(x)={({\left(-1\right)}^{j}/2^{n}n!)\bar{\nabla }}^{j-n}\Delta^{n}(g*e^{-r^{2}/2})(x)$. (c) Shows that the choice of the discrete operator has a big influence of the result.

 For implementing the discrete spherical derivatives, we propose to utilize central differences of 4th order accuracy for computing the partial derivatives (see Figure 2(b)). We observed that this scheme is a good tradeoff between computational complexity and accuracy. We experienced that the standard Laplace operator (considering a six voxel neighborhood) is numerically very unstable (even if double precision numbers are used!). Therefore, we propose the usage of the scheme depicted in Figure 2(a) which performed significantly better regarding numerical stability in our experiments. This is illustrated in Figure 3. As an example, we show the expansion images obtained via the proposed schemes together with the images obtained via a standard scheme. For comparison, we also show explicitly computed expansion images. The example illustrates that the ordinary Laplace operator leads to strong artifacts after a few number of applications.

### 5.2. Tensor Voting

 The Tensor Voting framework was originally proposed by [15] and has found several application in low-level vision in 2D and 3D. For example, it is used for perceptual grouping and extraction of line, curves, and surfaces. The key idea is to make unreliable measurements more robust by incorporating neighborhood information in a consistent and coherent manner. Following [4], the key expression that has to be computed is

(56) $$\begin{array}{c} U\left(r\right)=\int_{{\mathbb{R}}^{3}}V^{n(r^{\prime })}\left(r-r^{\prime }\right)m\left(r^{\prime }\right)dr^{\prime }, \end{array}$$

where **V** ^**n**^ : ℝ^3^ → *V*_*ℓ*_  is the voting field, *m* : ℝ^3^ → ℝ a scalar valued feature image giving evidence for the occurrence of the feature, and **n** : ℝ^3^ → ℝ^3^ the orientation of the feature of interest. In the following, we restrict ourselves to axial symmetric voting fields. Therefore, let *f*_*s*_ be a axial symmetric function, where the *z*-axis is the symmetry axis. Then, the voting field is

(57) $$\begin{array}{c} V^{n}\left(r\right)=\left(g_{n}f_{s}\right)\left(r\right), \end{array}$$

where *g*_**n**_ is a rotation such that the *z*-axis is mapped onto the axis defined by the normalized vector **n**. In [4], we have shown that (56) simplifies to

(58) $$\begin{array}{c} U\left(r\right)=\sum_{j=0}^{\infty }\;‍\sum_{k=-\ell }^{k=\ell }\left(E^{j+k}\;{\tilde{\circ }}_{\ell }\;A_{k}^{j}\right)\left(r\right), \end{array}$$

where

(59) $$\begin{array}{c} E^{j}\left(r\right)∶=m\left(r\right)Y^{j}\left(n\left(r\right)\right) \end{array}$$

are combined tensor-valued evidence images and

(60) $$\begin{array}{c} A_{k}^{j}\left(r\right)∶=a_{k}^{j}\left(r\right)Y^{j}\left(r\right) \end{array}$$

is the harmonic expansion of the voting field **V** ^**r**_*z*_^ steered in *z*-direction. The coefficients *a*_*k*_^*j*^(*r*) can be obtained by a projection on the tensorial harmonics

(61) $$\begin{array}{c} a_{k}^{j}\left(r\right)=N_{j,k}\int_{S_{r}^{2}}{\left(Z_{k0}^{j}\left(r\right)\right)}^{⊤}V^{r_{z}}\left(r\right)dr. \end{array}$$

Due to the symmetry of **V** ^**r**_*z*_^, only **Z**_*k*0_^*j*^ are involved. Further information concerning a practical point of view can be found in [14].

### 5.3. Nonlinear Covariant Filters

 In the following, we briefly show how to design trainable rotation covariant image filters which can be used for rotation invariant object or landmark detection. The idea is that expansion coefficients of a spherically expanded voting function are learned in a data driven way. The filter is mainly based on two steps. Rotation covariant image descriptors are densely computed in a voxel-by-voxel manner. Then, a weighted superposition of these image descriptors is used to form expansion coefficients of a spherical voting function. The expansion coefficients are formed such that each voting function votes for the presence or absence of landmarks or objects. The weights are found by a least square fit to a given training data set. For a fast implementation, we propose to use voting functions based on an expansion of spherical functions having a differential relationship in terms of spherical derivatives. In [18, 19], we used a spherical superposition of Gaussian windowed solid harmonics for representing the voting function. However, we are not restricted to them. For instance, we also can use the spherical plane-wave expansion leading to a voting function that is not only highly adaptable in angular direction, but also highly adaptable in radial direction, too; see the paper by [20]. The Fourier like voting function can be written as

(62) $$\begin{array}{c} V_{c}\left(r\right)=\int_{0}^{\infty }\sum_{j=0}^{\infty }V^{j}\left(c,k\right){•}_{0}B^{j}\left(r-c,k\right)dk, \end{array}$$

where **V** ^*j*^(*k*) ∈ *ℂ* ^2*j*+1^ are the expansion coefficients of the filter and *B* ^*j*^ are spherical Fourier basis functions known as Bessel functions (see (43)). The filter response is a saliency map representing the evidence for the presence or absence of objects. The saliency map is computed by collecting all contributions (votes) utilizing simple scalar valued convolutions. The explicit expression of the filter is

(63) ℋ{f}(r)∶=∫ℝ3Vc(r)dc=(∑j=0∞∫0∞(Bj(k)•~0Vj(k))dk)(r)(using  (33))=(∫0∞  B0(k)∗∑j∞∇jVj(k)dk)(r).

For implementation we use a band-limited expansion (up to order *N* ∈ *ℕ*) and only take a small set of frequencies (*k*_0_, ⋯*k*_*i*_ ⋯ , *k*_*i*_ ∈ ℝ) into account. We further make use of Gabor waves (see Theorem 16) to gain a filter that adapts and votes locally. In this case, the filter simplifies to

(64) $$\begin{array}{c} ℋ\left\{f\right\}\approx \sum_{i}B_{s}^{0}\left(k_{i}\right)*\sum_{j}^{N}\nabla_{j}V^{j}\left(k_{i}\right). \end{array}$$

**Figure 4:**
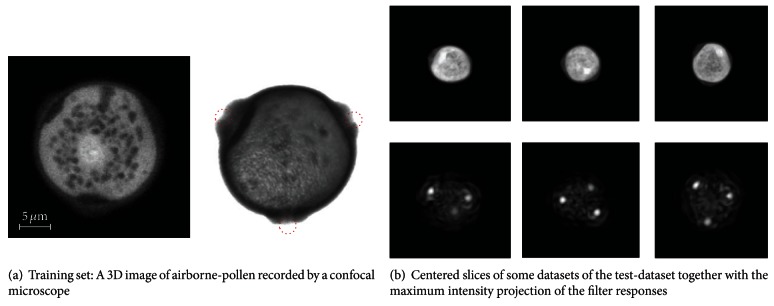
Filter response.

Trainable filters based on the Gabor waves have shown superior performance over the standard harmonic filters [20].  Figure 4 shows some qualitative results of an experiment where we detect the pores of airborne-pollen. The database contains 3D recordings of airborne-pollen acquired via a confocal laser scanning microscope. In Figure 4(a), we see the training image. The three porates are marked by red circles. In Figure 4(b), we exemplary show three datasets belonging to the test set together with the maximum intensity projection of the filter response.

### 5.4. Voxel-Wise Classification

**Figure 5:**
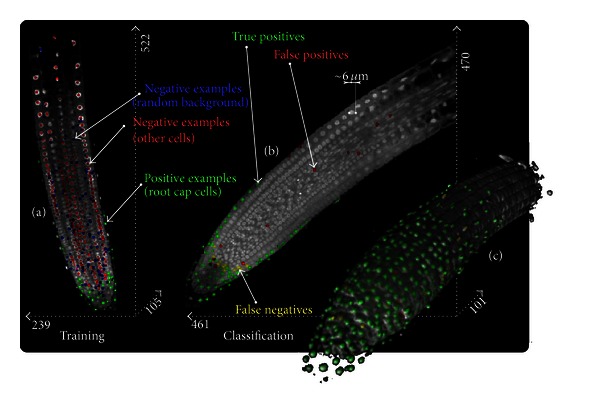
Voxel-wise classification of cells. For a voxel-wise classification, we first use a manually labeled image (a) for training a support vector machine (SVM) based on local rotation invariant image descriptors. Then, the SVM classifier is used to detect and classify cells in unclassified images (b). In (c) we depict an isosurface rendering of the classified root. Further details concerning the experiment can be found in [6].

 Especially in the field of biomedical imaging, the third dimension becomes more and more important due to the fact that organism can be studied in their natural constellation. Objects and organism can be located in any number at any position and, much more challenging, in any orientation. The third dimension does not only lead to larger datasets, but also the interrelation of neighboring intensity values becomes more complex. With a fast voxel-wise transformation of volumetric images into the harmonic domain, we are capable to compute rotation invariant image descriptors in an analytical way. In [6, 7], we used a fast Gabor transform to locally analyze images by decomposing local image patches into basic frequency components. For the experiments, we used confocal recordings of Arabidopsis root tips. We exemplary aimed at detecting differentiated cells located in the root cap. They morphologically differ from the other cells by their nonroundish shape. For this experiment, two datasets were used: one dataset for training and one dataset for evaluation. All cells (about 3600 in each root) were manually labeled by an expert. We transformed the Gabor expansion coefficients into invariant features utilizing the spherical tensor product; we combine the expansion coefficients corresponding to the same angular frequency, but not necessarily the same radial frequencies, whereas

(65) $$\begin{array}{c} c^{j}\left(k_{1},k_{2}\right)∶=\left(a^{j}\left(k_{1}\right)\;{•}_{0}\;a^{j}\left(k_{2}\right)\right), \end{array}$$

where *c* ^*j*^(*k*_1_, *k*_2_) ∈ *ℂ* are the rotation invariant image descriptors. It is worth mentioning that the combination of the same expansion coefficient coincides with the power-spectrum; namely, *c* ^*j*^(*k*) = (**a** ^*j*^(*k*)•_0_ **a** ^*j*^(*k*)) = ||**a** ^*j*^(*k*)||^2^. In Figure 5(a), we depict the center slice of the training data together with the training samples. Based on the rotation invariant image descriptors representing the training samples, an SVM classifier is trained. We used the SVM to classify test-set in a voxel-by-voxel manner (Figures 5(b) and 5(c)). We classed each voxel into root-cap cell or non-root-cap cell. For further details regarding the experiment, we refer to [6, 7].

### 5.5. DTI Processing

 Diffusion weighted magnetic resonance imaging (DWI) plays a substantial role in neuroscience and clinical applications. One field of interest is the investigation of the neuronal fiber architecture located in the brain white matter connecting different regions in the brain. The fibers themselves cannot be recorded directly. However, the data is usually recorded using the high angular resolution diffusion imaging (HARDI) technique [21], a specific kind of diffusion tensor imaging (DTI) technique. The resulting signal is an angular dependent, volumetric image. From such an image representation, the fiber architecture can be estimated (e.g., see [22]). Due to the angular dependency of HARDI signals, spherical harmonics are a common tool for signal representation. Therefore, in the context of DTI, there exist several applications worth considering spherical tensor algebra.

#### 5.5.1. Tissue Classification

 For the analysis of the fiber structure, a preprocessing step that identifies the brain white matter within the image is required. For group studies, the parcellation of the human brain into anatomical regions is of great interest. Preliminary results have been published in conference papers [23, 24].

We utilize the fact that the given recordings are tensor valued. We first transform the local measurements into the spherical harmonic domain (e.g., see [25]). Based on these rotation covariant image representations, we compute voxel-wise rotation invariant image features.

This is done by first comprising the voxels surrounding using the spherical down derivative operators. This can be seen as some kind of Taylor expansion of the given data. Then, we compute rotation invariant image features by computing the power spectrum of the resulting expansion coefficients. We finally use a random forest classifier [26] to learn the appearance of different kinds of brain regions and tissue types based on labeled training images. Such a parcellation might be for example, gray brain matter, white brain matter, and background signal. Qualitative results showing the resulting decisions of the random forest on an unclassified image are shown for the gray matter/white matter scenario in Figure 7. Furthermore, the votes for a certain class can be used as a kind of evidence value in further processing steps. Examples for the three classes background, white matter, and gray matter are depicted in Figure 8.

**Figure 6:**
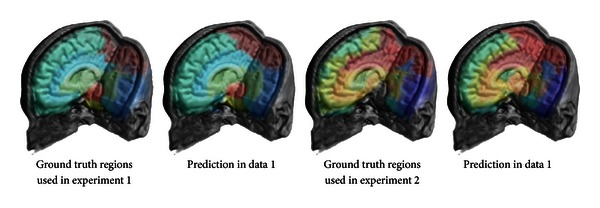
The ground truth regions that we used to train and evaluate our algorithm shown together with our algorithm's regions prediction. We can clearly see that our predictions are much more consistent with the data.

**Figure 7:**
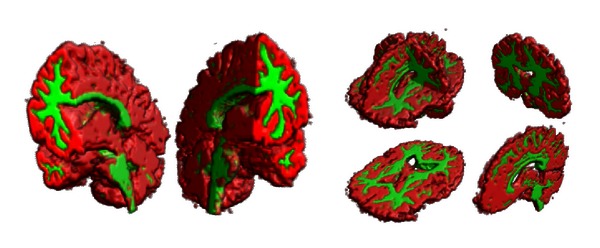
Isosurface showing the predictions for dataset 3 using GND and a random forest (RF) classifier. The classifier can distinguish between background, brain white matter (green), and gray matter (red).

**Figure 8:**
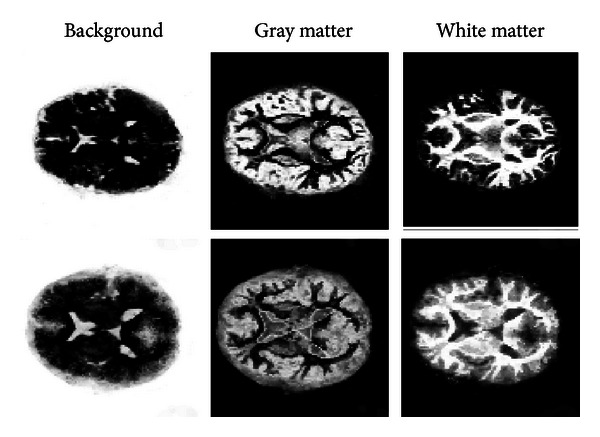
The confidence of the classifier represents the probability that a certain voxel belongs either to the background class, gray matter class, or the white matter class. The probability is represented by the intensity. A final decision is made by decision by majority (as shown in Figure 7).

**Figure 9:**
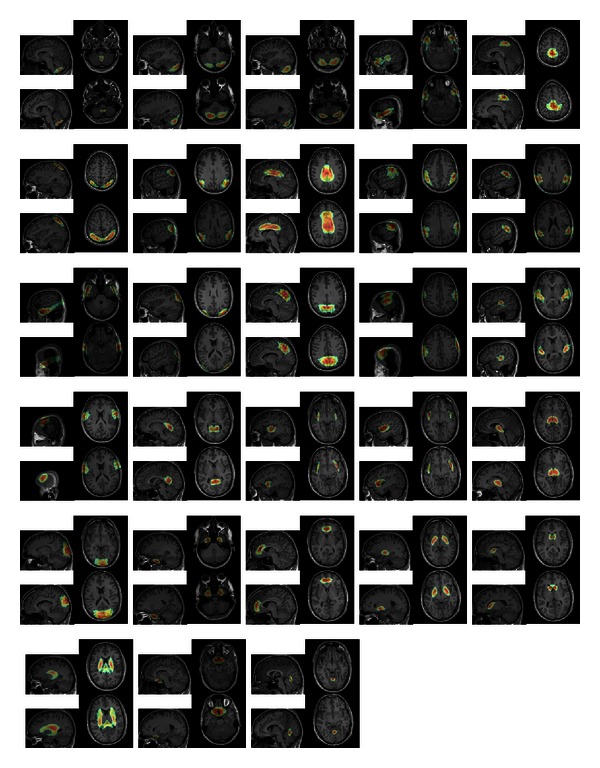
Heat maps representing the probability for all regions used in an experiment (continued in Figure 10).

**Figure 10:**
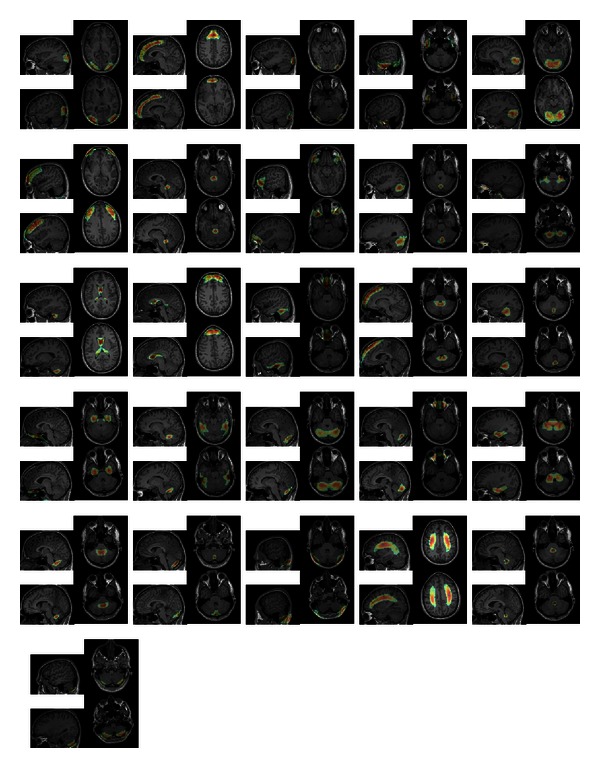
Heat maps representing the probability for all regions used an experiment (starting in Figure 9).

In Figures 9 and 10, we show the probability map of different kinds of brain regions that have been detected within unlabeled test images via a random forest classifier.  Figure 6 shows final predictions for one of the test sets.

#### 5.5.2. Unique Point-Landmark Detection

 Group studies often require the coregistration of images or partial image structures of different individuals. In such applications, the detection of characteristic landmarks is often an indispensable prerequisite.

Similar to [27], where features are used to find correspondences in scalar valued MR contrasts, we used tensor-based features in [28] offering a unique signature of a voxel's surrounding in tensor-valued HARDI signals. Thanks to these features, a large number of corresponding points can be reliably found in images of different individuals using a linear classifier. The features are computed in three steps. (1) We first entirely fit the HARDI signal to spherical harmonics. (2) The resulting fields are then efficiently expanded in terms of tensorial harmonics (Section 4) via tensor derivatives (see Section 3). (3) We obtain new covariant feature images which we use to form a trainable filter (see Section 5.3). The filter is used for the landmark detection task.

Second-order features which are sufficient for most applications are not providing enough information to solve the detection task in a human brain; they are invariant against reflection about an axis. Hence, they cannot distinguish the left and the right hemisphere. It is known that the spherical triple-correlation [29] yields complete rotation invariant features. Hence, they must solve this issue. Based on this idea we designed new 3rd order rotation invariant differential features fitting into our framework that are variant with respect to reflections about an axis. These features are additionally included in the harmonic filter framework. The triple product is given by

(66) ((baj1 ∘j baj2)∘j4baj3), j1+j2+j3+j4  is  odd,                   j4, j≤L,

where **b**_*a*_^*j*_1_^ ∈ *ℂ* ^2*j*_1_+1^, **b**_*a*_^*j*_2_^ ∈ *ℂ* ^2*j*_2_+1^, **b**_*a*_^*j*_3_^ ∈ *ℂ* ^2*j*_3_+1^ are the local tensorial harmonics expansion coefficients. A proof can be found in [28].

**Figure 11:**
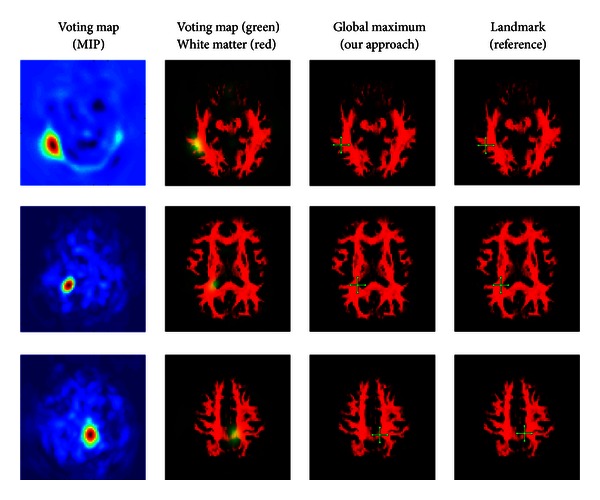
Differently weighted linear combinations of the feature images lead to different detection results.

**Figure 12:**
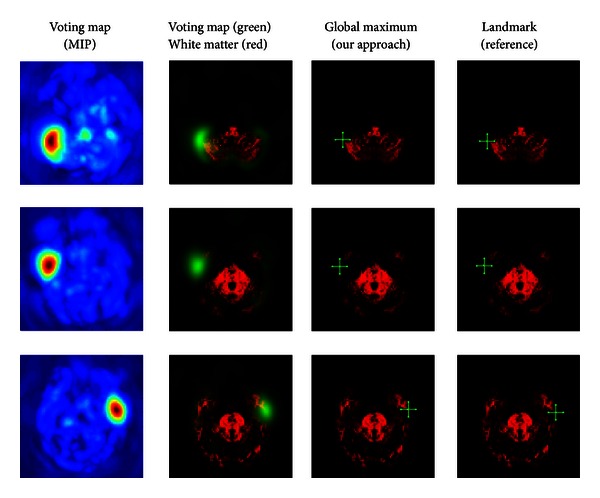
Differently weighted linear combinations of the feature images lead to different detection results.

**Figure 13:**
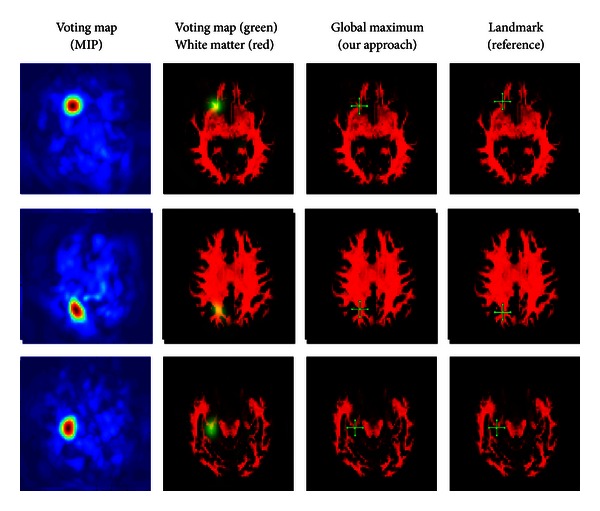
Differently weighted linear combinations of the feature images lead to different detection results.

**Figure 14:**
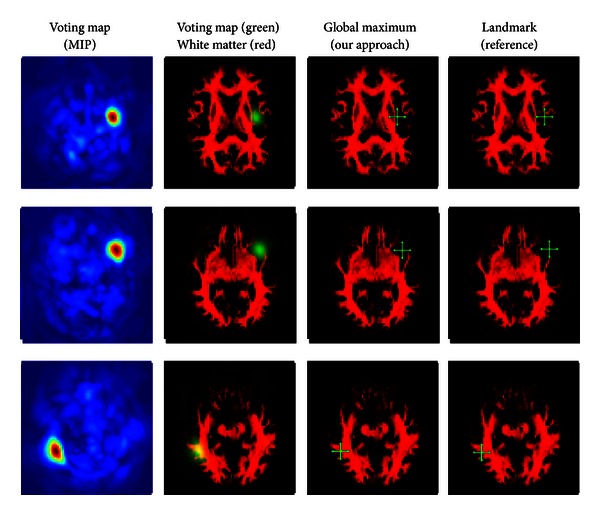
Differently weighted linear combinations of the feature images lead to different detection results.

The resulting filter has shown very promising results on a training set of 7 and a test set of 14 images. For the experiment, we placed about 20000 landmarks within the brain gray and white matter in an equidistant manner. For each dataset, the computation of the features and the detection of of all landmarks took about 5 minutes. We show some detection results in Figures 11, 12, 13, and 14.

## Appendices

### A. Spherical Harmonic Functions

The Schmidt seminormalized spherical harmonics *Y*_*m*_^*j*^ : *S*_2_ → *ℂ* are defined by

(A.1) $$\begin{array}{c} Y_{m}^{j}\left(r\right)∶=\sqrt{\frac{\left(j-m\right)!}{\left(j+m\right)!}}P_{j}^{m}\left(\cos\theta \right)e^{im\phi }, \end{array}$$

where *P*_*j*_^*m*^ are the with *m* associated Legendre polynomials of order *j* [30]. The spherical harmonics build a complete orthogonal basis for functions on the 2-sphere, whereas

(A.2) $$\begin{array}{c} \langle Y_{m}^{j},Y_{m^{\prime }}^{j^{\prime }}\rangle =\frac{\pi 4}{\left(2j+1\right)}\delta_{j,j^{\prime }}\delta_{m,m^{\prime }}. \end{array}$$

### B. Clebsch-Gordan Coefficients

Orthogonality

(B.1) ∑j,m〈jm ∣ j1m1,j2m2〉〈jm ∣ j1m1′,j2m2′〉  =δm1,m1′δm2,m2′,

(B.2) ∑j,m2j+12j1+1〈j1m1 ∣ jm,j2m2〉〈j1m1′ ∣ jm,j2m2′〉  =δm1,m1′δm2,m2′,

(B.3) ∑m=m1+m2〈jm ∣ j1m1,j2m2〉〈j′m′ ∣ j1m1,j2m2〉  =δj,j′δm,m′,

(B.4) ∑m1,m〈jm ∣ j1m1,j2m2〉〈jm ∣ j1m1,j2′m2′〉  =2j+12j2′+1δj2,j2′δm2,m2′.

Special values

(B.5) 〈ℓm ∣ (ℓ−λ)(m−μ),λμ〉  =(ℓ+mλ+μ)1/2(ℓ−mλ−μ)1/2(2ℓ2λ)−1/2,〈ℓm ∣ (ℓ+λ)(m−μ),λμ〉 =(−1)λ+μ(ℓ+λ−m+μλ+μ)1/2  ×(ℓ+λ+m−μλ−μ)1/2(2ℓ+2λ+12λ)−1/2.

Symmetry

(B.6) 〈jm ∣ j1m1,j2m2〉=〈j1m1,j2m2 ∣ jm〉,〈jm ∣ j1m1,j2m2〉=(−1)j+j1+j2〈jm ∣ j2m2,j1m1〉,〈jm ∣ j1m1,j2m2〉  =(−1)j+j1+j2〈j(−m) ∣ j1(−m1),j2(−m2)〉,〈jm ∣ j1m1,j2m2〉 =2j+12j2+1(−1)j1+m1〈j2m2 ∣ jm,j1(−m1)〉.

### C. Wigner D-Matrix

The components of **D**_*g*_^*ℓ*^ are written *D*_*mn*_^*ℓ*^. They are called the Wigner D-matrix. In Euler angles *ϕ*, *θ*, *ψ* in ZYZ-convention, we have

(C.1) $$\begin{array}{c} D_{mn}^{\ell }\left(\phi ,\theta ,\psi \right)=e^{im\phi }d_{mn}^{\ell }\left(\theta \right)e^{in\psi }, \end{array}$$

where *d*_*mn*_^*ℓ*^(*θ*) is the Wigner d-matrix which is real-valued. Relation to the Clebsch-Gordan coefficients:

(C.2) Dmnℓ=∑m1+m2=mn1+n2=nDm1n1ℓ1Dm2n2ℓ2〈lm ∣ l1m1,l2m2〉〈ln ∣ l1n1,l2n2〉,

(C.3) Dm1n1ℓ1Dm2n2ℓ2=∑l,m,nDmnℓ〈lm ∣ l1m1,l2m2〉〈ln ∣ l1n1,l2n2〉.

### D. Spherical Bessel Functions

The spherical Bessel functions *j*_*j*_ : ℝ_≥0_ → ℝ are related to the Bessel functions of the kind *J*_*ν*_ (e.g., see [30]) by $j_{j}(r)∶=\sqrt{\pi /2r}J_{j+1/2}(r)$ and are represented by the expansion

(D.1) $$\begin{array}{c} j_{j}\left(r\right)=r^{j}\sum_{m=0}^{\infty }\frac{{\left(-1\right)}^{m}}{2^{m}m!\left(2\left(j+m\right)+1\right)!!}r^{2m}, \end{array}$$

where

(D.2) $$\begin{array}{c} \int_{0}^{\infty }j_{j}\left(kr\right)j_{j}\left(k^{\prime }r\right)r^{2}dr=\frac{\pi }{2k^{2}}\delta \left(k-k^{\prime }\right). \end{array}$$

For the spherical Bessel functions, we have the following differential relations [30]:

(D.3) $$\begin{array}{c} \frac{\partial }{\partial r}\left[r^{-\nu }j_{\nu }\right]=-r^{-\nu }j_{\nu +1}, \end{array}$$

(D.4) $$\begin{array}{c} \frac{\partial }{\partial r}\left[r^{\nu +1}j_{\nu }\right]=r^{\nu +1}j_{\nu -1}. \end{array}$$

The Hankel Transform [31] (also known as Fourier-Bessel transform) of order *j* in terms of the spherical Bessel functions is given by

(D.5) $$\begin{array}{c} \alpha_{j}\left(k\right)=\int_{0}^{\infty }f\left(r\right)j_{j}\left(kr\right)r^{2}dr, \end{array}$$

and its corresponding inverse transformation is given by

(D.6) $$\begin{array}{c} f\left(r\right)=\frac{2}{\pi }\int_{0}^{\infty }\alpha_{j}\left(k\right)j_{j}\left(rk\right)k^{2}dk, \end{array}$$

which both are directly a result of (D.2).

### E. Plane Wave

Using the addition theorem of the spherical harmonics, we can express the spherical expansion of the plane wave (e.g., see [3, page 136]) in terms of the tensor product •_0_ leading to

(E.1) $$\begin{array}{c} e^{ik^{T}r}=\sum_{j}{\left(i\right)}^{j}\left(2j+1\right)j_{j}\left(kr\right)Y^{j}\left(r\right){•}_{0}Y^{j}\left(k\right), \end{array}$$

where *P*_*j*_ are the Legendre polynomials [30] of order *j* and **Y** ^*j*^ = (*Y*_−*j*_^*j*^,…, *Y*_*j*_^*j*^)^*T*^ the semi-Schmidt normalized spherical harmonics written as vector.

### F. Associated Laguerre Polynomials

The associated Laguerre polynomials [30] are defined by

(F.1) $$\begin{array}{c} L_{n}^{k}\left(x\right)=\sum_{i=0}^{n}{\left(-1\right)}^{i}\left(\begin{array}{c} n+k\\ n-i \end{array}\right)\frac{x^{i}}{i!}. \end{array}$$

The following 3-point-rule [30] is used in this work:

(F.2) $$\begin{array}{c} nL_{n}^{k}\left(x\right)=\left(n+k\right)L_{n-1}^{k}\left(x\right)-xL_{n-1}^{k+1}\left(x\right). \end{array}$$

We further need the the following differential equation [30]:

(F.3) $$\begin{array}{c} \frac{1}{m!}\frac{d^{m}}{dx^{m}}x^{k}L_{n}^{k}\left(x\right)=\left(\begin{array}{c} n+k\\ m \end{array}\right)x^{\left(k-m\right)}L_{n}^{\left(k-m\right)}\left(x\right). \end{array}$$

The polynomials *L*_*n*_^*k*^ and *L*_*n*′_^*k*^ are orthogonal over [0, *∞*) with respect to the weighting function *x* ^*k*^ *e* ^−*x*^ as

(F.4) $$\begin{array}{c} \int_{0}^{\infty }x^{k}e^{-x}L_{n}^{k}\left(x\right)L_{n^{\prime }}^{k}\left(x\right)dx=\frac{\Gamma \left(n+k+1\right)}{n!}\delta_{n,n^{\prime }}. \end{array}$$

For positive integers *n*, we have the following relation between the Gamma function and the double factorial [32, 33]:

(F.5) $$\begin{array}{c} \Gamma \left(n+\frac{1}{2}\right)=\frac{\left(2n-1\right)!!}{2^{n}}\sqrt{\pi }. \end{array}$$
